# Linkage of the CHHiP randomised controlled trial with primary care data: a study investigating ways of supplementing cancer trials and improving evidence-based practice

**DOI:** 10.1186/s12874-020-01078-9

**Published:** 2020-07-25

**Authors:** Agnieszka Lemanska, Rachel C. Byford, Clare Cruickshank, David P. Dearnaley, Filipa Ferreira, Clare Griffin, Emma Hall, William Hinton, Simon de Lusignan, Julian Sherlock, Sara Faithfull

**Affiliations:** 1 School of Health Sciences, Faculty of Health and Medical Sciences, University of Surrey, Guildford, GU2 7XH UK; 2 Data Science, National Physical Laboratory, Teddington, UK; 3 Nuffield Department of Primary Care Health Sciences, University of Oxford, Oxford, UK; 4 Clinical Trials and Statistics Unit, The Institute of Cancer Research, London, UK; 5 The Institute of Cancer Research and Royal Marsden NHS Trust, London, UK; 6 Royal College of General Practitioners (RCGP) Research and Surveillance Centre (RSC), London, UK; 7 School of Biosciences and Medicine, Faculty of Health and Medical Sciences, University of Surrey, Guildford, UK

**Keywords:** Primary care, Electronic health records, Data linkage, Randomised controlled trial

## Abstract

**Background:**

Randomised controlled trials (RCTs) are the gold standard for evidence-based practice. However, RCTs can have limitations. For example, translation of findings into practice can be limited by design features, such as inclusion criteria, not accurately reflecting clinical populations. In addition, it is expensive to recruit and follow-up participants in RCTs. Linkage with routinely collected data could offer a cost-effective way to enhance the conduct and generalisability of RCTs. The aim of this study is to investigate how primary care data can support RCTs.

**Methods:**

Secondary analysis following linkage of two datasets: 1) multicentre CHHiP radiotherapy trial (ISRCTN97182923) and 2) primary care database from the Royal College of General Practitioners Research and Surveillance Centre. Comorbidities and medications recorded in CHHiP at baseline, and radiotherapy-related toxicity recorded in CHHiP over time were compared with primary care records. The association of comorbidities and medications with toxicity was analysed with mixed-effects logistic regression.

**Results:**

Primary care records were extracted for 106 out of 2811 CHHiP participants recruited from sites in England (median age 70, range 44 to 82). Complementary information included longitudinal body mass index, blood pressure and cholesterol, as well as baseline smoking and alcohol usage but was limited by the considerable missing data. In the linked sample, 9 (8%) participants were recorded in CHHiP as having a history of diabetes and 38 (36%) hypertension, whereas primary care records indicated incidence prior to trial entry of 11 (10%) and 40 (38%) respectively. Concomitant medications were not collected in CHHiP but available in primary care records. This indicated that 44 (41.5%) men took aspirin, 65 (61.3%) statins, 14 (13.2%) metformin and 46 (43.4%) phosphodiesterase-5-inhibitors at some point before or after trial entry.

**Conclusions:**

We provide a set of recommendations on linkage and supplementation of trials. Data recorded in primary care are a rich resource and linkage could provide near real-time information to supplement trials and an efficient and cost-effective mechanism for long-term follow-up. In addition, standardised primary care data extracts could form part of RCT recruitment and conduct. However, this is at present limited by the variable quality and fragmentation of primary care data.

## Background

Randomised controlled trials (RCTs) are the gold standard of clinical research and are used to evaluate new treatments and improve current ones. However, RCTs can have limitations in informing evidence-based practice [1]. The information about the effectiveness and safety of a treatment is based on a population selected using strict eligibility criteria. Therefore, the results of RCTs may not be generalisable to the real-world clinical populations requiring treatments [2]. The complexity of cancer care increases with the rise in an ageing population, comorbidities and concomitant medications [3]. However, the evidence shows that people with advanced age or greater comorbidity are less likely to be recruited into clinical trials [4–7]. In addition, in pelvic radiotherapy commonly used for prostate cancer, side-effects can develop many years post-treatment but RCTs can be limited by a predefined length of follow-up and loss of participants to follow-up [8]. Furthermore, in RCTs focus is given to collecting data that is thought at the outset to be informative to the aims of a trial. This may change with evolving evidence, so methods may be required for extracting relevant information from routinely collated healthcare records.

In order to better understand the real-world effect or impact of new treatments, the results of RCTs should be supported with findings from other well-conducted studies. Observational studies using national registers (such as cancer or mortality data) and routinely collected data (both primary and secondary care), case studies and clinical reports could all be used to support RCTs in driving evidence-based practice [9–11]. This is to ensure that practice recommendations are based on real-world evidence. However, limitations have been identified with access, coverage (i.e. fragmented vs national) and coding accuracy of clinical concepts in routinely collected data in the English National Health Service (NHS) and worldwide [12–14]. Powell et al. (2017) investigated the feasibility of accessing a wide range of routinely collected clinical and non-clinical data to support RCTs [14]. However, due to fragmentation in coverage, they were not able to obtain primary care data for trial participants. In addition, large observational studies alone are still inadequate to drive practice change even if using methods such as propensity score matching of patients to limit the imbalance in known confounding variables between the investigated groups [15]. These methods have been shown to give misleading results in prostate cancer because they were unable to remove sources of bias in the effect estimates [16–18]. However, other approaches, that more closely match a clinical trial design in observational studies could ameliorate this [19].

There is a growing body of research that aims to widen the perspective by reusing and integrating other sources of data into RCTs [20–22]. This includes national data integration platforms that facilitate access to the relevant patient information to improve the speed and efficiency of clinical trials such as the NHS DigiTrials research hub in the United Kingdom (UK), the Big Data to Knowledge programme in the United States and the national research platform in Scotland [21] Research into the concept of data linkage to deliver more efficient trials is gaining momentum [23]. Internationally, the opportunities for linkage of routinely collected data and RCTs are increasing. This is through the creation of unique national identifiers such as the NHS number in England, the increasing use of electronic health records (EHR), and the creation of data infrastructures such as the Welsh Secure Anonymised Information Linkage databank [24], or the Clinical Practice Research Datalink in the UK [25]. In the United States, the challenges include costly and fragmented data infrastructures between public and private sectors [26]. However, an example of a successful model is the Swedish health system. It has complete and nationwide registers including cancer and death registers, and the systematic use of the national registration number that allows linkage across sectors [27].

The principal aim of this study was to assess the feasibility of linkage of RCTs to primary care data. The secondary aim was to assess the added value of utilising routinely collected data in the conduct and evaluation of results from RCTs. This study is unique and could have practical applications in methodologies for trial supplementation with primary care data. The study explored the important opportunities arising from trial linkage, including 1) the feasibility of linkage and 2) the added value in enhancing the conduct of RCTs e.g. in recruitment and long-term follow-up. The key contribution of this study is a set of recommendations on how the existing data infrastructure including collection of primary care records and data linkage could be strengthened to develop more accessible and comprehensive evidence that is implementable in real-world clinical practice.

## Methods

### The trial

A phase III multi-centre RCT of conventional versus hypofractionated high dose intensity modulated radiotherapy for prostate cancer (CHHiP) [28, 29] was used in this study. CHHiP (CRUK/06/16, REC reference 04/MRE02/10, registry number ISRCTN97182923) recruited 3216 participants with localised prostate cancer from 71 centres in the UK, Ireland, Switzerland, and New Zealand between 2002 and 2011. Men with prostate cancer were randomised to three different radiotherapy dose schedules: the standard schedule, or one of two hypofractionated and shorter schedules. The trial tested the hypothesis that hypofractionated radiotherapy schedules would improve the therapeutic ratio by either improving tumour control or reducing normal tissue side-effects, and demonstrated non-inferiority of one of the hypofractionated schedules in terms of biochemical/clinical failure with similar and low rates of toxicity [28, 29].

CHHiP prospectively collected longitudinal data on radiotherapy-related symptoms and toxicity reported both by participants and clinicians up to 5 years post treatment. A one-off morbidity questionnaire that included questions about ureteric obstructions, bowel strictures and bone fractures was collated at 10 years post treatment. Long-term follow-up of prostate specific antigen, prostate cancer recurrence and survival still continues. Clinician reported outcomes were collected prior to androgen deprivation therapy, prior to radiotherapy and at 6, 12, 18 and 24 months post radiotherapy with Late Effects on Normal Tissues: Subjective/Objective/Management (LENT/SOM) tool [30]. Three summary variables of LENT/SOM namely: “Any bladder urethra toxicity”, “Any rectal toxicity” and “Any sexual dysfunction” were used in regression to represent health domains that are most affected by prostate cancer, namely: bladder, bowel and sexual function respectively. In order to support linkage to routine data, NHS numbers (CHI numbers in Scotland) were also collected in CHHiP.

### The primary care database

Following research ethics and data governance approvals, CHHiP was linked to the primary care database, the Royal College of General Practitioners (RCGP) Research and Surveillance Centre (RSC). The RCGP RSC is a nationally representative network database [31], which has been collecting primary care data in England and monitoring disease trends for over 50 years [32]. At the time of the study, we used the December 2016 RCGP RSC database that comprised 164 practices and 1,275,174 registered adults (2.8% of the English population).

### Data access and approvals

Both datasets contain personal data and are therefore regulated by the European data protection law known as the General Data Protection Regulation and by the UK Data Protection Act 2018. Data are not shared publicly and the access is subject to approvals from a respective steering committee that governs each dataset. To gain access, to link and analyse the data and to protect patients, we needed to fulfil the information governance, practical and ethical requirements. An NHS ethics approval was obtained from the West of Scotland research ethics committee (REC1, 16/WS/0076). RCGP RSC research committee approved the protocol and the data request (RSC_0315). To obtain the CHHiP trial data, a formal data request was submitted together with the protocol describing the nature of the research and the extent of data required. The request was reviewed and approved by the Trial Management Group and independent Trial Steering Committee and a data sharing agreement was signed which described the condition for data release and how we fulfilled the requirements for secure data transfer, storage, archiving, publication and intellectual property.

### Data linkage

The RCGP RSC is an English primary care network, so only participants that were recruited to the CHHiP trial from sites in England (*n* = 2811) were used in this study. Using pseudonymised NHS numbers, primary care records were extracted for CHHiP participants and checked for quality. The primary care information included demographics such as ethnicity, socioeconomic status using the index of multiple deprivation (IMD), smoking and alcohol consumption statuses. The most recent entries from before recruitment to the trial were extracted. Comorbidities (cardiovascular and diabetes) and medications were extracted for two time periods, 1) any time before and 2) any time after recruitment to CHHiP (e.g. 1 - did they have diabetes before they entered CHHiP; and 2 - did they develop diabetes after they entered CHHiP). Medications included statins, anticoagulants, antihypertensives, heart and erectile dysfunction medications, and glucose-lowering drugs such as metformin. Cardiovascular health indicators such as body mass index (BMI, kg/m^2^), blood pressure (mmHg), glycated haemoglobin and lipids (including total cholesterol and high density lipoprotein) (mmol/l) were extracted longitudinally from 1 year before they entered to CHHiP. The process of pseudonymisation of NHS numbers, the linkage process and the choice of information extracted from primary care are described in detail in the published study protocol [33].

### Statistical analysis

Descriptive statistics were used to describe the linked sample of CHHiP participants. Mean and standard deviation were used for normally distributed variables. Median and interquartile range for non-normally distributed variables and count and percentage for categorical variables. Independent sample t-test, Mann-Whitney or chi-squared tests were used to test for differences in baseline characteristics between the linked sample and the unlinked CHHiP population to assess the representativeness of the linked sample. Multivariate logistic regression was used to explore the relationship of toxicity with comorbidities and medications, and the models were adjusted for age and randomisation group. Mixed-effects models were used to account for longitudinal changes in toxicity. Missing data in longitudinal profiles of LENT/SOM (47 (44%) missing pre-androgen deprivation therapy, 14 (13%) pre-radiotherapy, 8 (7%) at months 6 and 12, 11 (10%) at month 18, and 9 (8%) at month 24) were imputed using the last observation carried forward to minimise bias from missing data. If a pre-androgen deprivation therapy questionnaire was not completed it was imputed with the pre-radiotherapy data because androgen deprivation therapy was not used in all patients. Statistical significance was considered at the cut-off *p*-value of 0.01 to account for multiple comparisons. The RCGP RSC database was managed and extracted from the Structured Query Language 2016 database. Statistical analyses were performed in R version 3.0.2 (R Development Core Team, Austria).

## Results

### Data linkage

**Fig. 1:**
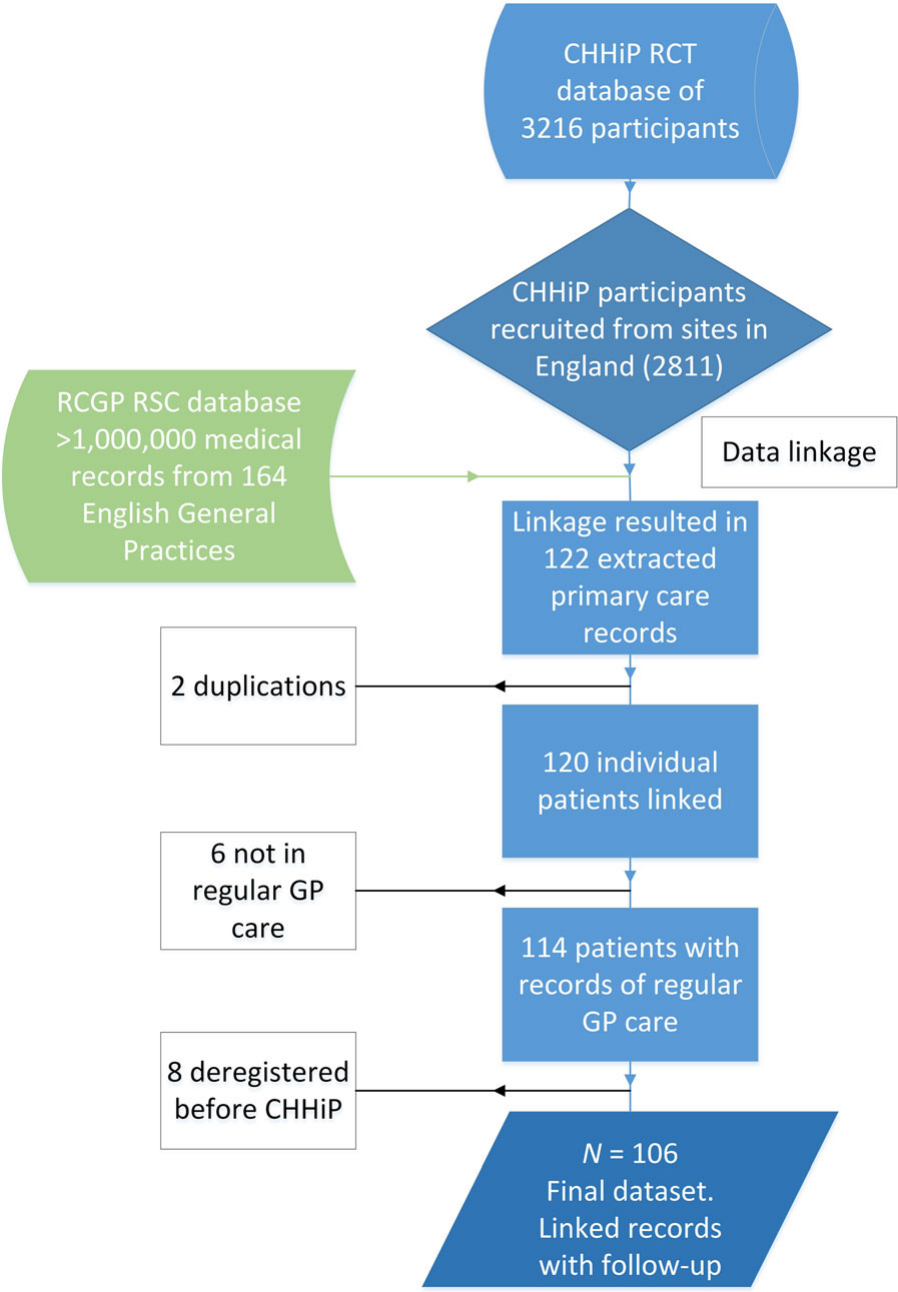
Consort flow of the linkage process demonstrating study cohort

RCGP RSC database was successfully linked to the CHHiP trial database using pseudonymised NHS numbers. Figure 1 shows the consort flow diagram of the linkage process and final study cohort. In total, 122 primary care EHRs were extracted for 120 out of 2811 CHHiP participants (two people had two EHRs because they moved from one practice to another practice within the network). This exceeded the estimation of 79 (the estimation based on RCGP RSC covering 2.8% of English population, 2.8% of 2811 is 79). However, after a closer inspection, 14 of the 120 linked records had to be excluded. Firstly, six EHRs were for participants with a ‘*temporary’* or ‘*walk-in’* registration status meaning that they were not receiving regular primary care. For example, an EHR would only contain a record of an emergency prescription or a one-off procedure for an acute injury e.g. broken leg. Secondly, a further eight EHRs were excluded for participants who deregistered from a practice in the RCGP RSC network before they entered the CHHiP trial. These participants registered with another practice outside of the RCGP RSC network, so no EHRs were retrieved for the period of the trial. This resulted in a final dataset of *N* = 106 linked sample (3.8% of the trial population) available for statistical analysis.

### Supplementing baseline trial data

Baseline characteristics collected within the CHHiP trial of the linked sample are provided in Table 1. The median age was 70 years (range: 44 to 82; IQR: 65 to 74). The linked sample was representative of the overall CHHiP population. The additional baseline characteristics extracted for CHHiP participants from the primary care records are presented in Table 2. This shows that 64 participants (93% of those whose ethnicity was recorded) were white, 4 (6%) were black and 1 (1%) was of Asian ethnicity. Participants from the least deprived socioeconomic backgrounds (IMD quintile = 5) formed the highest percentage (37, 40%), while 7 (8%) were from the most deprived socioeconomic backgrounds (IMD quintile = 1). Data also show that 12 (27%) men were obese (BMI ≥ 30 kg/m^2^) in the year they entered the trial, 5 (6%) actively smoked and 25 (29%) were drinking alcohol at or above the hazardous level. However, because we extracted the most recent data from prior to trial, the information on alcohol and smoking could potentially be from years before the trial and could have changed over time for some participants. So, for example, smoking data, was from within 1 year for 50 men (63% of 79 whose smoking data were recorded). The median for the whole sample was 1 year but the range was 0 to 27 years prior to CHHiP. In this sample, the proportion of missing / unavailable data in primary care records was considerable. Ethnicity was not recorded for 37 (35%) men, IMD score for 13 (12%), smoking status and alcohol consumption status were not recorded for 27 (25%) and 20 (19%) men respectively.

**Table 1:** Baseline characteristics of the linked sample of trial participants as collected in the CHHiP trial. The linked sample is representative of the overall trial population [28]. *P*-values are calculated using independent sample t-test, Mann–Whitney or chi-squared tests. IQR, interquartile range

	CHHiP unlinked sample *n* = 3110	Linked sample *N* = 106	*P*-value
Age at randomisation to CHHiP (years)			
Median (IQR)	69 (64–73)	70 (65–74)	0.13
Range	48–85	44–82	
Randomisation, *n* (%)			0.04
Group 1 (standard schedule, 74Gy/37f)	1041 (33)	24 (23)	
Group 2 (hypofractionated schedule 1, 60Gy/20f)	1029 (33)	45 (42)	
Group 3 (hypofractionated schedule 2, 57Gy/19f)	1040 (33)	37 (35)	
Clinical T stage, *n* (%)			0.10
T1	1125 (36)	45 (42)	
T2	1718 (55)	48 (45)	
T3	264 (9)	13 (12)	
Missing or unknown	3 (< 1)	0 (0)	
Type of androgen deprivation therapy, *n* (%)			0.66
Luteinising-hormone-releasing hormone plus short-term anti-androgen	2608 (84)	92 (87)	
Bicalutamide (150 mg)	393 (13)	10 (9)	
Other	9 (< 1)	0 (0)	
None	86 (3)	4 (4)	
Time from start of androgen deprivation therapy to radiotherapy, median (IQR), (weeks)	16 (14–20)	17 (14–21)	0.29
Past clinical history of comorbidity, *n* (%)			
Diabetes	333 (11)	9 (8)	0.57
Hypertension	1238 (40)	38 (36)	0.47
Inflammatory bowel disease	122 (4)	2 (2)	0.42
Symptomatic haemorrhoids	204 (7)	5 (5)	0.58
Previous pelvic surgery, *n* (%)	244 (8)	8 (7)	1.00
Previous transurethral resection of the prostate, *n* (%)	243 (8)	16 (15)	0.01
Alpha-blockers or anticholinergic drugs at trial entry, *n* (%)	363 (12)	12 (11)	1.00
Year of entry to the CHHiP trial, n (%)			0.65
Year 2–3	106 (3)	2 (2)	
Year 4–5	258 (8)	11 (10)	
Year 6–7	521 (17)	17 (16)	
Year 8–9	1338 (43)	41 (39)	
Year 10–11	886 (28)	35 (33)

**Table 2:** Baseline characteristics of the linked sample of CHHiP participants based on additional information extracted from primary care data

Baseline characteristic	*n* (% of recorded)	Mean (SD) of recorded	Missing *n* (% of 106 linked sample)
Ethnicity			37 (35)
White	64 (93)		
Black	4 (6)		
Asian	1 (1)		
Index of multiple deprivation, quintile			13 (12)
1 (≥ 33.881, most deprived)	7 (8)		
2	14 (15)		
3	15 (16)		
4	20 (22)		
5 (≤ 8.372, least deprived)	37 (40)		
Body mass index (kg/m^2^)		28 (5)	61 (57)
Obesity (body mass index ≥30)	12 (27)		
Systolic blood pressure (mmHg)		141 (20)	48 (45)
Diastolic blood pressure (mmHg)		78 (11)	48 (45)
Total cholesterol (mmol/l)		4.7 (1.0)	68 (64)
High-density lipoprotein (mmol/l)		1.3 (0.3)	73 (69)
Smoking status			27 (25)
Active smoker	5 (6)		
Ex-smoker	43 (54)		
Non-smoker	31 (39)		
Alcohol consumption			20 (19)
Non-drinker	10 (12)		
Safe	51 (59)		
Hazardous	21 (24)		
Alcoholism	4 (5)		

### Supplementing trial data on comorbidities and medications

Table 3 reports comorbidities and concomitant medications recorded in EHRs separately for two periods: 1) before and 2) after enrolment to the CHHiP trial. 12 (11%) trial participants developed diabetes (8 within 3 years after enrolment to the CHHiP trial) and 9 (8%) developed hypertension (5 of them within 3 years after enrolment). Cardiovascular and diabetes medications were not recorded in the CHHiP trial but were extracted from the primary care data. These included angiotensin converting enzyme inhibitors, statins, metformin, aspirin, phosphodiesterase type 5 inhibitors and rectal steroids. Data show that 22 (20.8%), 40 (37.7%), 7 (6.6%), 32 (30.2%), 20 (18.9%) and 7 (6.6%) of participants respectively commenced these medications before trial entry, and 15 (14.2%), 25 (23.6%), 7 (6.6%), 12 (11.3%), 26 (24.5%) and 17 (16.0%) additional participants were prescribed them after trial entry.

**Table 3:** Information on comorbidities and prescription medications recorded for the linked sample. Column 1: information recorded in the CHHiP trial at baseline (pre-radiotherapy), Column 2: information in primary care before enrolment to the CHHiP trial and Column 5: information in primary care after enrolment to the CHHiP trial. Date of the trial enrolment was defined as the start date of androgen deprivation therapy. Columns 3 and 4: information not recorded in each of the databases respectively

	Information recorded at baseline in the CHHiP trial *n* (%)	Information recorded in primary care before enrolment to CHHiP *n* (%)	Not recoded in the trial *n* (%)	Not recorded in primary care *n* (%)	Information recorded in primary care after enrolment to CHHiP *n* (%) (new diagnosis)
Past clinical history of comorbidity					
Diabetes	9 (8)	11 (10) (type1–1, type2–10)	2 (2)	0	12 (11) (all participants type2)
Hypertension	38 (36)	40 (38)	14 (13)	12 (11)	9 (8)
Inflammatory bowel disease	2 (2)	Not extracted	–	–	–
Symptomatic haemorrhoids	5 (5)	5 (5)	5 (5)	5 (5)	14 (13)
Previous pelvic surgery / prostatectomy	8 (7)	14 (13)	11 (10)	5 (5)	3 (3)
Previous transurethral resection of the prostate	16 (15)	52 (49)	38 (36)	2 (2)	3 (3)
Medication					
Alpha-blocker or anticholinergic	12 (11)	9 (8)	8 (7)	11 (10)	16 (15)
Angiotensin converting enzyme inhibitors	Not recorded	22 (20)	22 (21)	–	15 (14)
Statins	Not recorded	40 (38)	40 (38)	–	25 (24)
Metformin	Not recorded	7 (7)	7 (7)	–	7 (7)
Aspirin	Not recorded	32 (30)	32 (30)	–	12 (11)
Phosphodiesterase type 5 inhibitors	Not recorded	20 (19)	20 (19)	–	26 (25)
Rectal steroids	Not recorded	7 (7)	7 (7)	–	17 (16)

**Fig. 2:**
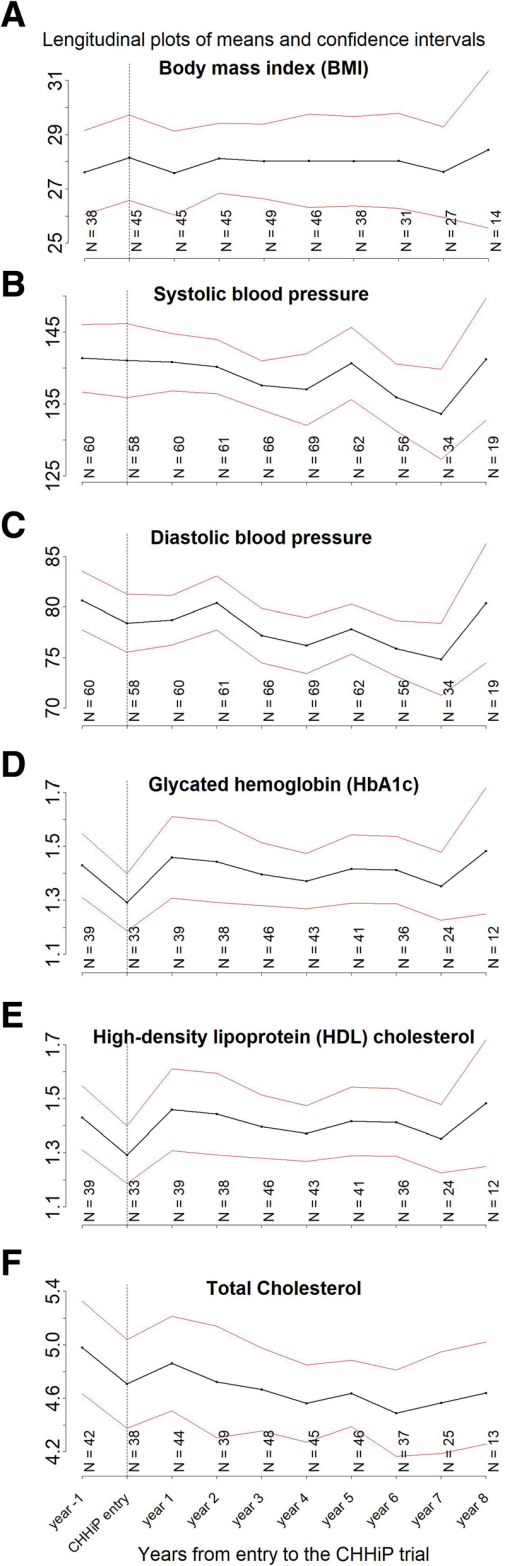
Longitudinal profiles of means and confidence intervals for body mass index (BMI), systolic and diastolic blood pressure, glycated haemoglobin (HbA1c) and high-density lipoprotein (HDL) and total cholesterol. N numbers represent the number of participants with data available at each point in time

In addition, from the primary care data, it was possible to monitor changes over time in cardiovascular health and diabetes indicators. The longitudinal profiles starting from 1 year before entry to CHHiP and presented for up to 8 years after (due to the increasing number of missing data), are presented in Fig. 2 (with the numbers of participants for whom data was recorded). In the year of entry to CHHiP, BMI was only recorded for 45 (42%) men. The other clinical tests including BP and cholesterol are also not recorded for a considerable number of men. Statistical analysis of how cardiovascular health varied over time pre and post cancer diagnosis, and treatment was limited by inadequate statistical power due to the small linked sample.

### Agreement between data sources

Anticholinergic and alpha-blocking medications were recorded at baseline in CHHiP for 12 (11%) men but only 1 of these was also identified in primary care records. In total, 9 (8%) men were identified in primary care records as having prescriptions for these medications at some time before they entered the CHHiP trial and out of the 9, 6 (6%) had evidence of ongoing prescriptions after trial entry. A further 16 (15%) men commenced these medications after trial entry.

There were also some discrepancies between CHHiP and primary care data in recording comorbidities. In the case of diabetes, there was a relatively good agreement with 9 (11%) men with diabetes recorded in both sources and only 2 (2%) cases that were missed in CHHiP baseline data. A close inspection of diagnosis dates from primary care data revealed that these two men were diagnosed with diabetes at 39 and 92 days before entry to CHHiP. However, the discrepancies in recording hypertension were more challenging. In the linked sample, 38 (36%) participants were recorded as having a history of hypertension at the point of trial entry, whereas primary care records indicated incidence of 40 (38%) prior to trial entry, with 14 (13%) cases potentially missed in CHHiP and 12 (11%) not recorded in primary care data. An additional 9 (8%) men were diagnosed with hypertension after enrolling to CHHiP.

### The association of comorbidities and medications with radiotherapy outcomes

In Table 4 the results of exploratory regression analysis are presented to investigate the association of comorbidities and medications with bladder, bowel and sexual toxicity recorded with LENT/SOM tool over time. The small linked sample limits the statistical power of these analyses. However, the association of diabetes and metformin (both *p* = 0.008) with sexual toxicity is worth noting.

**Table 4:** Exploratory analysis using mixed-effects logistic regression of longitudinal bladder, bowel and sexual toxicity recorded with LENT/SOM in CHHiP, to analyse the association with comorbidities and medications recorded in primary care at entry to CHHiP. Separate models are fitted for each of the independent variables (comorbidities and medications yes / no [reference]) accounting for age at entry and trial randomisation arm. *statistically significant at a level of *p* < 0.01 (cut-off used in this study). ACE, angiotensin converting enzyme; LENT/SOM, Late Effects on Normal Tissues: Subjective/Objective/Management

Dependent variable	Independent variable	Coefficient estimate	Lower 95% CI	Upper 95% CI	OR	*P*-value
LENT/SOM bladder toxicity (yes / no)	Diabetes	0.70	−0.54	1.94	2.02	0.27
	Hypertension	0.46	−0.55	1.46	1.58	0.38
	Metformin	0.54	−1.20	2.28	1.72	0.54
	Aspirin	0.20	−0.88	1.28	1.22	0.72
	Statins	−0.14	−1.04	0.76	0.87	0.76
	ACE inhibitors	−0.12	−1.27	1.03	0.89	0.84
	PDE5 inhibitors	0.99	0.02	1.95	2.68	0.05
LENT/SOM bowel toxicity (yes / no)	Diabetes	0.39	−0.79	1.57	1.48	0.52
	Hypertension	−0.01	−0.90	0.87	0.99	0.98
	Metformin	−0.05	−1.50	1.40	0.95	0.95
	Aspirin	0.26	−0.66	1.18	1.30	0.58
	Statins	−0.91	−1.73	−0.08	0.40	0.03
	ACE inhibitors	0.53	−0.53	1.58	1.69	0.33
	PDE5 inhibitors	0.14	−0.78	1.06	1.15	0.76
LENT/SOM sexual toxicity (yes / no)	Diabetes	−1.87	−3.26	−0.480	0.15	0.008*
	Hypertension	0.29	−0.65	1.220	1.33	0.55
	Metformin	−3.08	−5.36	−0.800	0.05	0.008*
	Aspirin	−0.49	−1.46	0.480	0.62	0.33
	Statins	−0.42	−1.24	0.400	0.66	0.32
	ACE inhibitors	−0.98	−2.04	0.080	0.37	0.07
	PDE5 inhibitors	−0.27	−1.14	0.600	0.76	0.54

## Discussion

### The value of the linked resource

This study is important methodologically because it demonstrates that linkage of cancer clinical trials and primary care data is feasible, and could be a valuable way of supplementing trials with comorbidities and medications for supporting conduct and evaluation of RCTs. However, in this study this was limited by the fragmentation and varying quality of primary care data, including considerable missing data for some data items. These findings are not unique to the UK setting and similar challenges could be observed in the United States where data are fragmented between public and private sectors [26]. Unique patient identifiers that are used successfully in many high income countries such as the European member states [34] can offer a part of the solution through opportunities to trace patients across the healthcare systems. However, countries need to invest resources into innovative infrastructures that integrate disparate parts of healthcare systems, and into standardised and interoperable software. In addition they need to clearly consider the new information governance (including patient safety), ethical and policy challenges that come along with these opportunities [35, 36]. The information on comorbidities is important because the literature shows that comorbidities can result in worse radiotherapy outcomes such as sexual or physical functioning [37, 38], but points to the positive effects of some cardiovascular medications [39–42]. The evidence is still inconclusive and there is a need for more research in this area.

The rates of missing data for some data items were considerable, so if looking for information within a small time window (thus clinically relevant) for example BMI at the time of trial entry, the missingness could present a challenge. However, data could still have value for tracking change over time and picking up trends related to an event. The standards of recording clinical information such as comorbidities and medications in primary care have improved significantly over recent years. In the UK, primary care has been computerised since the late 1990s and the introduction of a pay-for-performance system for chronic disease management in 2004 resulted in much more consistent recording of comorbidities in EHR as seen in this study with diabetes. This reflects the increasing value of primary care data for research [43]. Other countries such as Germany, Netherlands, Australia and New Zealand have also highly computerised primary care but in the United States and Canada, primary care so far is less computerised [44].

### Barriers and facilitators to a linkage study

Funding for this study was granted in mid-2016 at which point the team commenced drafting an application for ethical approval and both data sharing requests (described in the Data access and approvals section). Approvals have taken a considerable amount of effort and time (around a year) to set up. The study protocol was published in 2017 [33]. Once all the permissions were in place, we transferred and linked data. The linkage using NHS numbers is a simple (methodologically) procedure. However, the next considerable challenge was to curate and extract prostate cancer and radiotherapy-related health outcomes from primary care data. They are recorded in primary care using different coding systems and varying terminology. Therefore, ontologies were developed to systematically and transparently map the clinical concepts across the primary care data. The lists of clinical codes used to extract the outcomes are available on request. The first two outputs were conference papers. One at the International Population Data Linkage Network, Canada 2018 [45] and the second at the Public Health England, Cancer Data Conference, 2018 [10.13140/RG.2.2.10494.43849].

### Study limitations

The analyses of the value of primary care records are limited in this study by the small linked sample that may not be representative of the whole RCGP RSC population. At present there is no national primary care database in the UK. The RCGP RSC database is one of the largest but at the time of the study it included only 2.8% of the English population [31]. It was a model database to test the feasibility of the linkage process and investigate the value of the linked resource. The additional value of the RCGP RSC was that they had the appropriate data infrastructure, while other databases that we approached could not support bespoke linkage. We expected a limited number of EHRs extracted for CHHiP patients and a larger database would be needed for an adequate trial coverage. Since then, the RCGP RSC network has grown considerably and now includes more than 500 practices so an updated analysis would likely return a larger sample.

Due to the nature of the clinical cohort (prostate cancer) this study included only men, and therefore the results cannot be interpreted in the light of possible gender disparities e.g. in the usage of healthcare systems or in the availability of primary care data. Another limitation was a considerable amount of missing data. CHHiP recruited between 2002 and 2011, and the quality of primary care data has improved significantly since then, so the percentage of missing data at present may be smaller than that shown in this study. The small linked sample also limits statistical analysis of the association of comorbidities with radiotherapy outcomes. However, some comorbidities such as diabetes have been identified as important targets for future research. In addition, the exclusion of 14 (13%) participants with incomplete EHRs i.e. those who de-registered from the network before they entered the CHHiP trial and those with a ‘*temporary’* or ‘*walk-in’* registration status is a limitation because it could introduce bias. When designing the study, a decision was made not to limit data linkage based on primary care registration status. This was not to exclude participants in error due to potentially miscoded registration status. This was a relatively small-scale study so a manual quality inspection of EHRs was possible. However, because we have found no such errors, we recommend that participants with other than the ‘*registered’* registration statuses should automatically be excluded.

## Recommendations

### Quality and coverage issues

#### Quality of information

In most countries where primary care is a gatekeeper to the healthcare system, primary care physicians will be the first point of call for patients. This means that all consultations that are not accident, emergency or specialist are delivered in primary care. Secondary care feeds back information to primary care which should also be coded. A large proportion of prescribing is also carried out in primary care. Therefore, nearly all health-related events and medications are recorded and can be extracted from EHRs. However, the downsides of routine primary care data are that they are collected during routine consultations or based on letters from hospitals so the quality depends greatly on the key data being coded by clinicians often limited by time [46]. Therefore, to support the use of EHR in supplementing RCTs, the consistency and standards of recording needs to improve [47].

In the UK, there are guidelines on recording cancer diagnosis information in primary care [48, 49]. However, we recommend that work is carried out to equip secondary care with relevant lists of primary care clinical codes for outcomes (so that they can be included in letters to primary care) to standardise recording. Above all, the adoption across the NHS of the single clinical terminology called SNOMED-CT (systematised nomenclature of medicine - clinical terms) recommended in the ‘Personalised Health and Care 2020’ policy framework [50] would allow accurate recording and flow of clinical information across healthcare sectors.

#### Fragmentation in coverage of primary care databases

In the UK, 98% of the population is registered with primary care and has a unique NHS number (CHI number in Scotland). However, in this study it was not possible to extract primary care records for all CHHiP participants. There are other large, nationally representative databases apart from RCGP RSC but there is no mandatory, national primary care database, and currently only around 10% of the nearly 10,000 practices contribute data so the resource is fragmented [51]. To improve quality of data and research, a national primary care database, governed by NHS Digital is warranted.

### Potential opportunities

#### Supplementing RCTs with comorbidities and medications

The evidence base is evolving, and comorbidities other than those considered at the outset of a trial may become important (e.g. affecting outcomes of radiotherapy). Therefore, data linkage and EHRs could in the future be used to provide information on comorbidities and prescription medications to allow more efficient conduct of clinical trials and to support secondary analyses.

Comorbidities and prescription medications, if recorded, are usually collected at baseline in trials (to rule out contraindications) with ongoing collection likely to be limited in large phase III non-registration trials. Design features of clinical trials, such as randomisation and sufficient sample size, reduce the risk of imbalance between treatment groups in both known and unknown baseline factors thus minimising bias when comparing the groups. However, trial participants can develop new comorbidities and commence medications after enrolment, and this may be important in terms of the ongoing safety of patients (in which case it would be collected as part of the trial) or for the interpretation and evaluation of outcomes from clinical trials. In the future, linkage with primary care could facilitate this, but data infrastructures and standards for recording of outcomes data in primary care need to improve. Efficient conduct of RCTs.

Targeted screening strategies and standardised primary care data summaries could support efficient conduct of clinical trials. For example, their value is increasingly being recognised in facilitating recruitment to trials (prospective and retrospective) by enabling screening and eligibility checks as part of the process [22]. They could also support baseline data collection with standardised extracts feeding into trials. This would reduce burden to participants and hospital based research teams.

#### Enhancing long term follow-up in trials

Linkage to primary care could provide near real-time information which can supplement key trial outcomes data and cost-effective mechanisms for long-term follow-up beyond RCTs. Primary care data are a rich resource with breadth and depth of clinical information recorded for people with cancer over time. Hence the opportunities should be recognised for secondary analysis to support primary results of RCTs and to test secondary hypotheses.

## Conclusions

This is the first UK study to link a large prostate cancer RCT to primary care. Therefore, this study is significant in providing evidence on the feasibility of linkage of RCTs to routinely collected data. In addition, this study’s unique contribution is in presenting evidence related to the value of the linked resource in enhancing the conduct of RCTs. Although more research is needed, these findings are not limited to the UK setting and therefore the proof-of-concept methodology presented here could be positioned as the future state-of-the-art methodology for trial supplementation. Information that is routinely recorded in primary care databases is a rich resource that could potentially be used in the future to support evaluation of outcomes of RCTs in real-world populations enhancing generalisability of trials. Primary care data could also be used for long-term follow-up beyond the duration of trials. This is important because with improvements in cancer diagnoses and treatments, patients survive many years and can develop late toxicity which may be difficult to investigate and potentially underreported. In addition to the enhanced follow-up, another opportunity is in extracting standardised primary care data summaries that would include comorbidities and medications. This could support screening and recruitment processes, and supplement baseline data collection in RCTs [52–54]. However, more research is needed to better understand the opportunities and challenges associated with the use of EHR in supplementing RCTs.

## Data Availability

The data that support the findings of this study are available from the Institute of Cancer Research Clinical Trials and Statistics Unit and the Royal College of General Practitioners Research and Surveillance Centre with restrictions that apply to the availability of data, which were used under license for the current study, and so are not publicly available. Data are however available upon reasonable request, with permission of the Royal College of General Practitioners Research and Surveillance Centre and the Institute of Cancer Research in accordance with the data sharing policies.

## Abbreviations

BMI: Body mass index
CHHiP: Conventional versus Hypofractionated High dose IMRT for Prostate cancer (a phase III multi-centre RCT)
CHI: Community health index (Scotland)
EHR: Electronic health records
IMD: Index of multiple deprivation
IQR: Interquartile range
LENT/SOM: Late Effects on Normal Tissues: Subjective/Objective/Management
NHS: National Health Service
RCGP: Royal College of General Practitioners
RCT: Randomised controlled trial
RSC: Research and Surveillance Centre
UK: United Kingdom

## References

[1] Sibbald B, Roland M (1998). Understanding controlled trials. Why are randomised controlled trials important?. BMJ (Clinical research ed.).

[2] Stuart EA, Bradshaw CP, Leaf PJ (2015). Assessing the generalizability of randomized trial results to target populations. Prev Sci.

[3] Sarfati D, Koczwara B, Jackson C (2016). The impact of comorbidity on cancer and its treatment. CA Cancer J Clin.

[4] Malatestinic W (2017). Characteristics and medication use of psoriasis patients who may or may not qualify for randomized controlled trials. J Manag Care Spec Pharm.

[5] Hutchinson-Jaffe AB (2010). Comparison of baseline characteristics, management and outcome of patients with non-ST-segment elevation acute coronary syndrome in versus not in clinical trials. Am J Cardiol.

[6] Dalela D (2017). Generalizability of the prostate cancer intervention versus observation trial (PIVOT) results to contemporary north American men with prostate cancer. Eur Urol.

[7] Kennedy-Martin T (2015). A literature review on the representativeness of randomized controlled trial samples and implications for the external validity of trial results. Trials.

[8] Sanson-Fisher RW (2007). Limitations of the randomized controlled trial in evaluating population-based health interventions. Am J Prev Med.

[9] Krauss A (2018). Why all randomised controlled trials produce biased results. Ann Med.

[10] Black N (1996). Why we need observational studies to evaluate the effectiveness of health care. BMJ.

[11] Booth CM, Tannock IF (2014). Randomised controlled trials and population-based observational research: partners in the evolution of medical evidence. Br J Cancer.

[12] Campbell JR (1997). Phase II evaluation of clinical coding schemes: completeness, taxonomy, mapping, definitions, and clarity. CPRI Work Group on Codes and Structures. J Am Med Inform Assoc.

[13] de Lusignan S (2012). Call for consistent coding in diabetes mellitus using the Royal College of General Practitioners and NHS pragmatic classification of diabetes. Inform Prim Care.

[14] Powell GA (2017). Using routinely recorded data in the UK to assess outcomes in a randomised controlled trial: the trials of access. Trials.

[15] Ad N (2015). Practice changes in blood glucose management following open heart surgery: from a prospective randomized study to everyday practice. Eur J Cardiothorac Surg.

[16] Hadley J (2010). Comparative effectiveness of prostate cancer treatments: evaluating statistical adjustments for confounding in observational data. J Natl Cancer Inst.

[17] Zumsteg ZS, Zelefsky MJ. Improved survival with surgery in prostate cancer patients without medical comorbidity: a self-fulfilling prophecy? Eur Urol. 2013;64(3):381–3. https://www.thelancet.com/journals/lanonc/article/PIIS1470-2045(16)30102-4/fulltext. 10.1016/j.eururo.2013.05.037 23746719

[18] Tree AC, van As NJ, Dearnaley DP (2016). Re: Christopher J.D. Wallis, Refik Saskin, Richard Choo, et al. Surgery versus radiotherapy for clinically-localized prostate cancer: a systematic review and meta-analysis. Eur Urol 2016;70:21–30. Eur Urol.

[19] Dickerman BA (2019). Avoidable flaws in observational analyses: an application to statins and cancer. Nat Med.

[20] Kilburn LS (2017). Can routine data be used to support cancer clinical trials? A historical baseline on which to build: retrospective linkage of data from the TACT (CRUK 01/001) breast cancer trial and the National Cancer Data Repository. Trials.

[21] Gray CM, Wyke S, Zhang R (2018). Long-term weight loss following a randomised controlled trial of a weight management programme for men delivered through professional football clubs: the Football Fans in Training follow-up study.

[22] Mc Cord KA (2018). Routinely collected data for randomized trials: promises, barriers, and implications. Trials.

[23] Lewsey JD (2000). Using routine data to complement and enhance the results of randomised controlled trials. Health Technol Assess.

[24] Lyons RA (2009). The SAIL databank: linking multiple health and social care datasets. BMC Med Inform Decis Mak.

[25] Padmanabhan S (2019). Approach to record linkage of primary care data from Clinical Practice Research Datalink to other health-related patient data: overview and implications. Eur J Epidemiol.

[26] Bradley CJ (2010). Health services research and data linkages: issues, methods, and directions for the future. Health Serv Res.

[27] Adami H-O (1996). A paradise for epidemiologists?. Lancet.

[28] Dearnaley D (2016). Conventional versus hypofractionated high-dose intensity-modulated radiotherapy for prostate cancer: 5-year outcomes of the randomised, non-inferiority, phase 3 CHHiP trial. Lancet Oncol.

[29] Wilkins A (2015). Hypofractionated radiotherapy versus conventionally fractionated radiotherapy for patients with intermediate-risk localised prostate cancer: 2-year patient-reported outcomes of the randomised, non-inferiority, phase 3 CHHiP trial. Lancet Oncol.

[30] LENT SOMA tables. Radiother Oncol. 1995;35:17–60. 7569012

[31] Correa A, et al. Royal College of General Practitioners Research and Surveillance Centre (RCGP RSC) sentinel network: a cohort profile. BMJ Open. 2016;6:e011092. 10.1136/bmjopen-2016-011092. 10.1136/bmjopen-2016-011092 PMC4838708 27098827

[32] de Lusignan S (2017). RCGP Research and Surveillance Centre: 50 years’ surveillance of influenza, infections, and respiratory conditions. Br J Gen Pract.

[33] Lemanska A (2017). Linking CHHiP prostate cancer RCT with GP records: a study proposal to investigate the effect of co-morbidities and medications on long-term symptoms and radiotherapy-related toxicity. Tech Innov Patient Support Radiat Oncol.

[34] Mills S (2019). Unique health identifiers for universal health coverage. J Health Popul Nutr.

[35] Holm S, Ploug T (2017). Big data and health research-the governance challenges in a mixed data economy. J Bioeth Inq.

[36] Vayena E (2018). Digital health: meeting the ethical and policy challenges. Swiss Med Wkly.

[37] Vissers PA (2016). The impact of having both cancer and diabetes on patient-reported outcomes: a systematic review and directions for future research. J Cancer Surviv.

[38] Skwarchuk MW (2000). Late rectal toxicity after conformal radiotherapy of prostate cancer (I): multivariate analysis and dose-response. Int J Radiat Oncol Biol Phys.

[39] van der Veen SJ (2015). ACE inhibition attenuates radiation-induced cardiopulmonary damage. Radiother Oncol.

[40] Wedlake LJ (2012). Evaluating the efficacy of statins and ACE-inhibitors in reducing gastrointestinal toxicity in patients receiving radiotherapy for pelvic malignancies. Eur J Cancer.

[41] Kollmeier MA (2011). Improved biochemical outcomes with statin use in patients with high-risk localized prostate cancer treated with radiotherapy. Int J Radiat Oncol Biol Phys.

[42] Ostrau C (2009). Lovastatin attenuates ionizing radiation-induced normal tissue damage in vivo. Radiother Oncol.

[43] de Lusignan S, Van Weel C (2006). The use of routinely collected computer data for research in primary care: opportunities and challenges. Fam Pract.

[44] Jha AK (2008). The use of health information technology in seven nations. Int J Med Inform.

[45] Lemanska, A., et al., Extracting primary care records for prostate cancer patients in the CHHiP multicentre randomised control trial: a healthcare data linkage study. 2018. doi.org/10.23889/ijpds.v3i4.741.

[46] Liyanage H (2018). Ontologies in big health data analytics: application to routine clinical data. Stud Health Technol Inform.

[47] Khan NF (2011). Long-term health outcomes in a British cohort of breast, colorectal and prostate cancer survivors: a database study. Br J Cancer.

[48] The Transforming Cancer Services Team for London (TSCT), Tower Hamlets CCG and Tower Hamlets Clinical Effectiveness Group (2019). Guidance on clinical coding of cancer patients in primary care.

[49] Bhuiya A (2017). London cancer and Macmillan cancer support: a guide to quality coding and safety netting in the context of cancer.

[50] National Information Board (2014). Personalised health and care 2020. Using data and technology to transform outcomes for patients and citizens: a framework for action.

[51] Vezyridis P, Timmons S (2016). Evolution of primary care databases in UK: a scientometric analysis of research output. BMJ Open.

[52] Kopcke F (2013). Secondary use of routinely collected patient data in a clinical trial: an evaluation of the effects on patient recruitment and data acquisition. Int J Med Inform.

[53] Cornelius VR (2018). Automated recruitment and randomisation for an efficient randomised controlled trial in primary care. Trials.

[54] Brooks CJ (2009). Use of a patient linked data warehouse to facilitate diabetes trial recruitment from primary care. Prim Care Diabetes.

